# Amyloidogenic Intrinsically Disordered Proteins: New Insights into Their Self-Assembly and Their Interaction with Membranes

**DOI:** 10.3390/life10080144

**Published:** 2020-08-08

**Authors:** Federica Scollo, Carmelo La Rosa

**Affiliations:** 1 Department of Biophysical Chemistry, J. Heyrovský Institute of Physical Chemistry, Academy of Sciences of the Czech Republic, Dolejškova 3, 18223 Prague 8, Czech Republic; federica.scollo@jh-inst.cas.cz; 2 Dipartimento di Scienze Chimiche, Università degli Studi di Catania, Viale Andrea Doria 6, 95125 Catania, Italy

**Keywords:** intrinsically disordered proteins, lipid, membrane, CMC, amyloids, toxicity, lipids, protein-transport, lipid-assisted protein transport, symmetry-breaking

## Abstract

Aβ, IAPP, α-synuclein, and prion proteins belong to the amyloidogenic intrinsically disordered proteins’ family; indeed, they lack well defined secondary and tertiary structures. It is generally acknowledged that they are involved, respectively, in Alzheimer’s, Type II Diabetes Mellitus, Parkinson’s, and Creutzfeldt–Jakob’s diseases. The molecular mechanism of toxicity is under intense debate, as many hypotheses concerning the involvement of the amyloid and the toxic oligomers have been proposed. However, the main role is represented by the interplay of protein and the cell membrane. Thus, the understanding of the interaction mechanism at the molecular level is crucial to shed light on the dynamics driving this phenomenon. There are plenty of factors influencing the interaction as mentioned above, however, the overall view is made trickier by the apparent irreproducibility and inconsistency of the data reported in the literature. Here, we contextualized this topic in a historical, and even more importantly, in a future perspective. We introduce two novel insights: the chemical equilibrium, always established in the aqueous phase between the free and the membrane phospholipids, as mediators of protein-transport into the core of the bilayer, and the symmetry-breaking of oligomeric aggregates forming an alternating array of partially ordered and disordered monomers.

## 1. Introduction

Proteins and lipids are vital elements of every form of life. Proteins, as the most abundant macromolecules of living beings, have many functions ranging from the catalysis of essential biochemical reactions to the transport of nutrients. Similarly, lipids are involved in energy storage, and they play fundamental structural roles as well, being the main component of the cell membranes. The intrinsically disordered proteins (IDPs) are present as highly dynamic ensembles, characterized by significantly variable atom positions over time. Their structural adaptability provides IDPs unique functional capabilities that cannot be accomplished by folded proteins. Due to their delicate biological tasks, not surprisingly, IDPs are also involved in a large number of human diseases and represent one of the most attractive (and challenging) drug targets of the last decade. IDP-associated disorders, including Type II Diabetes Mellitus (T2DM), Alzheimer’s (AD), and Parkinson’s Diseases (PD). All are characterized by the conversion of peptides or proteins from their soluble functional states into fibrillar aggregates, showing a cross-beta super-secondary structure, termed “amyloid”. Investigations aimed to explain the observed correlation of amyloid plaques and disease have led to what is known as the “amyloid hypothesis” [1]. 

Nonetheless, more recently, it has become evident that the amyloid hypothesis must be amended. This readjustment stems from the evidence that amyloid fibers themselves are not a direct cause of disease pathology. For instance, the correlation of the extent of the Amyloid β peptide (Aβ) deposits with dementia in AD patients is poor. Similarly, amyloid deposition in T2DM is evident in about 90% of patients [2]. However, this fails to explain the fact that the remaining 10% of patients do not present significant amyloid deposition [3]. Such observations suggest that intermediate structures of amyloid formation could be more relevant to the insurgence of the pathology. To date, the mechanism by which these amyloid intermediates cause cytotoxicity and disease has not yet been clarified [4]. One of the major hypotheses is that amyloid peptides cause membrane perturbation through changes in membrane fluidity, amyloid peptide-induced ion channel-like formation, or free radical production and simultaneous lipid peroxidation. They may affect the plasma membrane as well as internal membranes, such as the mitochondrial ones. 

Many amyloid-forming peptides are generally amphipathic structures. Therefore, they may have the capacity to insert into membranes where they may eventually generate, by a self-assembling process, protein-stabilized pores (poration) [5,6], lay on one leaflet of membranes (carpeting), or remove the lipid components from the bilayer by a detergent-like mechanism [7]. Nowadays, there is no universal consensus about which perturbations are relevant to the diseases.

Likewise, lipids do not merely provide the matrix where proteins are embedded, but can also actively participate in the regulation of protein activity, trafficking, and localization. Moreover, lipids act as a medium, and their physical–chemical properties, like nano-segregation (raft), thickness, fluidity, or curvature, regulate protein functions. The characterization of lipid–protein interactions is, to our knowledge, a key factor of the organizational principles of cell membranes. In this frame, the great complexity of cell membranes emerges, and it is evident how challenging it is to study, at an atomic level, the fundamental interactions between lipid–lipid and lipid–proteins in the cell. To overcome this complexity, many researchers applied a “bottom up” strategy. A variety of membrane model systems were developed. Multi-lamellar vesicles (MLVs), large unilamellar vesicles (LUVs), giant unilamellar vesicles (GUVs), small unilamellar vesicles (SUVs), supported lipid bilayers (SLBs), black lipid membranes (BLMs), and Blodgett–Langmuir monolayer films are a few examples. These simplified model systems were used to investigate the fundamental forces driving lipid–lipid and lipid–proteins interactions. One popular area of investigation involves the studies on the interaction between model membrane and amyloidogenic or intrinsically disordered proteins; these studies are relevant in order to find the molecular path of IDPs toxicity and thus to design an effective drugs treatment for T2DM, AD, and PD, which affect a significant percentage of the world’s population. In this review, we discuss these topics according to a historical approach and report an on latest novel hypotheses, such as lipid-assisted protein transport and the breaking-symmetry of oligomeric aggregates forming an alternating array of partially ordered and disordered monomers. 

**Figure 1:**
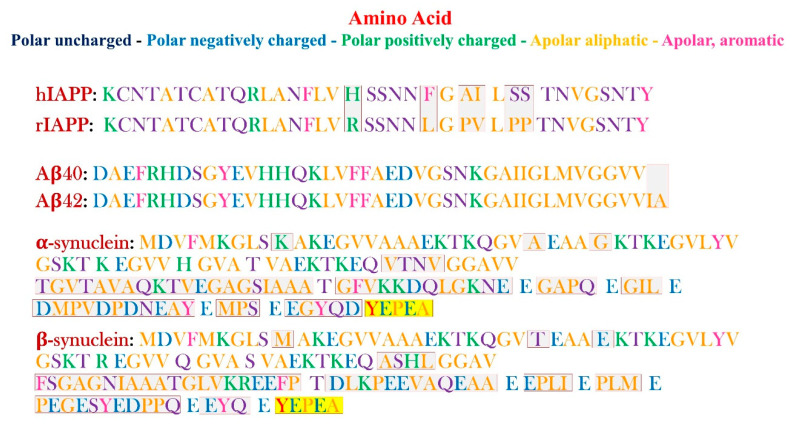
Schematic representation of the primary sequences of hIAPP, rIAPP, Aβ_(1-40)_ and Aβ_(1-42)_, α and β-synuclein (respectively, from the top to the bottom). The different amino acid residues are pictured in different colors, according to the legend (on the top right of the figure). The differences between the h- and r-IAPP, Aβ_(1-40)_ and Aβ_(1-42)_, α and β-synuclein, respectively, are highlighted by red boxes for the first two pairs, and by red boxes for the last couple of proteins.

Figure 1 shows the amino acid sequences of the IAPP (rat and human), Aβ (40 and 42), and synuclein (α and β), described in more detail in the following chapters.

## 2. Amyloidogenic Proteins

### 2.1. Amyloid-β Peptide

The Aβ is a 39–43 amino acid amphiphilic polypeptide, characterized by a hydrophobic region (C-terminal) and a hydrophilic one (N-terminal). At the physiological pH, the global net charge is -3, since the primary sequence of Aβ consists of six negatively charged residues (Asp1, Glu3, Asp7, Glu11, Glu22, Asp23) and three positively charged residues (Arg5, Lys16, Lys28). In vivo, the Aβ-peptide is expressed and originated from a larger transmembrane glycoprotein named amyloid precursor protein (APP) and composed of 770 amino acids [8,9,10].

The link between APP and AD has already been ascertained, and it can be traced in the proteolytic action of APP by β-secretase, which provokes the release of a soluble extracellular domain (aAPPβ) and intracellular peptide’s fragments [11]. Those are later on cleaved by the γ-secretase to originate Aβ and the APP intracellular domain [12,13].

The most common amyloidogenic forms are 40 and 42 amino acids in length, named Aβ_(1-40)_ and Aβ_(1-42)_. They differ because of the two final residues. The Aβ_(1-42)_ primary structure includes two residues, isoleucine and alanine, following the two ending valine residues, as schematically shown in fig. 1. Both the peptides have been found as two major components of the amyloid plaques characterizing the AD disease, although the smallest peptide is less prone to the aggregation. The latter is believed to be less amyloidogenic, and this is the reason why the Aβ_(1-42)_ is the most predominant component of the plaques [14]. Indeed, it has been shown that an increase in Aβ_(1-42)_ might play a key role in the insurgence of AD [15].

However, due to their low solubility, both the peptides are prone to aggregation. This feature is associated with neuronal degeneration, a typical manifestation of dementia characterizing the patients affected by AD [8]. Many in vivo and in vitro studies have aimed at understanding both the toxicity and the interaction of the peptide with model membranes. In fact, the fibrillation is observed at µM concentration in vitro either for Aβ_(1-40)_ or Aβ_(1-42)_, even though the latter aggregates faster compared to the other variant. Aβ_(1-40)_ is present in the cerebrospinal fluid (CSF) in nM concentration [8,16], and it is neurotoxic at approximately 10 µM concentrations [17]. Moreover, recent studies have pointed out the repetition of the sequence GXXXG as responsible for toxicity and pore formation [18,19,20].

### 2.2. IAPP

Islet amyloid polypeptide (IAPP), also called Amylin, is a hormone normally co-secreted with the insulin by the β-cells of the pancreas [21,22,23,24]. The protein was isolated for the first time from the pancreas tissues of T2DM patients in 1987 by Cooper and Westermark, independently [25,26]. In its soluble form, IAPP is an unstructured, C-terminally amidated peptide, composed of 37 amino acids, with a disulfide bond established between residues 2 and 7 [27]. Its function is still under intense debate, but it is thought to be involved in the regulation of insulin secretion, glucose metabolism, and homeostasis [28,29]. Due to its capacity to form pores in the plasma membrane and those organelles involved in protein synthesis and secretory pathways (i.e., the endoplasmatic reticulum) [30], hIAPP aggregates might be toxic to pancreatic β-cells. Indeed, in T2DM, hIAPP deposits as amyloid fibers are found in the extracellular spaces of the pancreas, together with the depletion of β -cells. Human amylin (hIAPP) has a global net charge of + 3, while for the rat-variant, this is +4. For the two forms, it is possible to identify some differences in the primary structure. In particular, the sequence varies for six residues (five of which are located in the 20-29 region [31,32]). 

More specifically, the non-amyloidogenic rIAPP contains three prolines located in positions 25, 28, and 29, differently than the hIAPP [33]. Moreover, the His18 hIAPP is replaced by Arg in rIAPP, and Leu23 and Val26, respectively, substitute Phe23 and Ile26 in hIAPP [34,35]. The distinct sequences are shown in Figure 1.

Interestingly, Amylin exists in millimolar concentration in the secretory granules [36], and its predisposition to aggregation is inhibited by the interaction with insulin, but not with proinsulin [37,38]. Whereas in vitro, hIAPP aggregates and forms amyloid under the physiological condition from 10 μM upwards [39,40], spanning from the minutes to hours timescale, while the rIAPP does not [21]. The inability of r-Amylin to form fibrils might be due to the three prolines situated in the central region. In fact, proline is considered to be a β-structure breaker, since it affects the order structure typical of the fibril formation, simply destabilizing the amyloid fibrils [20,21,22].

### 2.3. Synuclein

α-synuclein has been found as a major component of the intracellular deposits known as Lewy bodies (LB) [41], composed of proteinaceous aggregates [42], by membranes fragments and distorted organelles [43], often associated with dementia and PD. However, the key components of the LB is still under debate. Their form and whether there is a concomitant and synergic role, played by all the components, in the formation of LB is still unknown [44]. This protein, composed of 140 amino acids (~14.5 kDa), is expressed in the brain and is predominantly located in synaptic terminals [42,45]. The intracellular concentration has been estimated to be less than 30–60 μM [46,47]. Its function in non-pathological conditions has not yet been clearly understood; amidst all, it is thought to mediate the vesicular trafficking at the membrane [48]. Indeed, this lipid–protein interaction is responsible for many biological functions, such as synaptic plasticity or neurotransmitter release [49], but it can also be the driving force of the protein’s aggregation propensity. Additionally, the self-assembly of the SNARE complex, either in vitro and in vivo, is influenced by synuclein through the formation of multimers at the surface of the synaptic vesicles [50]. 

However, it is generally acknowledged that this protein is involved in PD-associated neurodegeneration [51]. Among the typical clinical symptoms, these include the impairment of cognitive abilities and neuropsychiatric dysfunctions, such as tremor, depression, anxiety, and psychosis [52,53]. At the physiological level, a significant loss of dopaminergic neurons in the *substantia nigra* has been observed. Since these neurons are involved in the motor processes control, this might be the cause of the debilitation motor, and cognitive neurological degeneration characteristic of the patients [54]. The protein is mostly unstructured. Together with the IAPP and Aβ, it is classified as an IDP since, in solution, it encompasses a large ensemble of conformations. Its self-assembly and its conversion into toxic oligomers and as a result, amyloid fibers are distinctive of the pathogenesis of PD. As a matter of fact, α-synuclein’s first 95 residues go through a significant conformational transition upon binding with lipid membranes, passing from random-coil, the typical secondary structure of the monomeric disordered form, to predominantly an α-helical structure [55,56]. The extent of this transition is influenced by the nature of the lipids constituting the membrane [57] and the protein-to-lipid-ratio [58,59]. The aggregation mechanism is also influenced by the membrane composition [60]. However, it has been shown that α-synuclein forms β-sheets-rich oligomers and subsequently, amyloid fibers [61,62] at meaningfully higher concentrations compared to the physiological levels. Therefore, the fibrillation in vivo might be due to synuclein overexpression [63], to the inhibition of its degradation [64], as well as its concentration may reach a higher level because of non-specific molecular crowding [65,66].

α-synuclein belongs to a family that includes β and γ-synuclein as well. All these proteins have in common the lack of a well-defined secondary and tertiary structure, which confers them the “IDPs label.” Interestingly, it has been shown that the β-synuclein inhibits the aggregation of α-synuclein [67]. In addition, several studies highlighted the less propensity to form amyloid fibers of the β compared to the α-form [67,68,69]. The latter is a natively unfolded 134 residues protein, which shares 78% of the primary structure’s homology with the amyloidogenic α-synuclein (Figure 1) [68]. Notably, the lack of 11 specific amino acids in the central region might be the causal factor for its non-amyloidogenic features [70], because this affects the interruption of the helical structure related to this specific region. The N-terminal region (residues 60–95), common to both proteins, contains a series of KTKEGV repetitive sequences [71]. These are characteristic of amphipathic helices in apolipoproteins, which mediate the lipid binding.

### 2.4. Prion

Prion is a family of proteins related to the transmissible spongiform encephalopathies (TSEs), also known as prion diseases. These are fatal neurodegenerative disorders affecting both animals and humans (e.g., Creutzfeldt–Jakob disease). According to the “protein-only hypothesis,” the etiology of the diseases is associated to an abnormal conversion of the prion protein [72] that accumulates by forming plaques in the brain. Specifically, two isoforms exist, both made of 209 residues: the physiological cellular one (PrP^C^) and the disease-associated infectious form (PrP^Sc^) [73]. In mammalians, the prion replicates as a consequence of the recruitment of the normal PrP^C^ isoform, and its conversion into the PrP^Sc^ is somehow stimulated [74].

The PrP^C^ is a cell surface glycosylphosphatidylinositol-anchored glycoprotein, with a well folded α-helical C-terminal domain and a flexible and unstructured N-terminal fragment [75]. Its secondary structure was characterized using Fourier transform infrared spectroscopy and circular dichroism, and it was shown to be mainly consisting of α-helix (42%) and no β-sheets (3%). In contrast, the β-sheet content of PrP^Sc^ was significantly higher (43%), and the α-helix amounted to 30%. Proteinase digestion cleaved a 67 amino acids N-terminal, giving rise to PrP 27-30, which was characterized by a 54% β-sheets and a 21% α-helix content [76]. 

More specifically, three α-helices and two short β-strands constitute the globular C-terminal domains, all together forming a unique β1–α1–β2–α2–α3, in which the β-strands are arranged together, establishing an anti-parallel β-ribbon [75]. The N-terminus and the C-terminus are linked by a highly conserved middle region composed of four positively charged lysine residues and a hydrophobic region. This region is located from positions 112 to 135, and it has a high tendency to acquire a β-sheet structure [77,78]. Several studies have pointed out the involvement of this hydrophobic region into the conformational change propagation of the PrP, occurring when the PrP^Sc^ acts as the seed and template for the transition of Pr^PC^ into PrP^Sc^. This is thought to be one of the leading causes of the insurgence of prion disease [75]. In addition, nuclear magnetic resonance (NMR) studies in solutions have been used to assess the monomeric structure of mouse [79], Syrian hamster [80,81], murine [82] and human prion proteins [83]. Prion protein self-assembling seems to play a pivotal role in the abnormal conversion process of PrP^C^ into PrP^SC^ [84]. Indeed, in vitro aggregation occurs through a classical nucleation and growth process, showing a lag phase of hours at the micro or submicromolar concentrations [85,86] which is similar to the concentration levels found in healthy brains [87]. In these conditions, the protein forms amorphous aggregates [76], amyloid-like fibril structures [88], or two-dimensional crystals [89], while in vivo, it aggregates in the form of plaques or deposits [90].

Prion diseases are associated with an abnormal conformational transition involving the prion protein, and they are known to affect mammals. The different mechanical behavior of two mammalians, human (HuPrP) and Syrian hamster (ShaPrP), and two non-mammalian prions, chicken (ChPrP) and turtle (TuPrP), were assessed by steered molecular dynamics simulations (MD), performed on the globular domains of the four proteins [91]. In mammalian prions, higher resistance to external stretching forces and an earlier occurrence of irreversible events were observed. The different unfolding profile of mammalian prions, ascribable to the intramolecular interactions involving helix 1 with helix 3, implicates the existence of metastable non-native states, which may prompt abnormal pathways of protein misfolding and aggregation. [54]. 

## 3. Amyloidogenic Proteins and Model Membranes

### 3.1. Interaction with Model Membranes

The role of lipid membrane–amyloid interaction is certainly crucial in the corresponding pathogeneses. Indeed, it is generally approved that amyloidogenic proteins can be absorbed on the surface, insert into the membranes, or even mimic the cell-penetrating peptides’ behavior [92]. Plenty of studies have been performed to elucidate the mechanisms at the basis of the aforementioned interactions, toward either the prevention or the treatment of these widespread diseases. This feature, together with the oligomerization process, is common to all the protein described in this review. The oligomerization tendency has the potential to generate protein-stabilized pores in the context of a membrane [93]. As previously underlined, using a bottom-up approach is of fundamental importance to study the interplay between membranes and the amyloidogenic proteins, using both experimental and computational methods. 

It has been found out that many factor play a decisive role in these mechanisms, such as membrane composition, net charge surface, protein-to-lipid ratio, pH and ionic strength, the concentration of the peptide, presence of ions in solution [40,58,94,95,96,97,98,99,100,101,102,103,104].

Among all the possible lipids combinations, cholesterol, sphingomyelin, and gangliosides, not necessarily together, play a pivotal role in these phenomena. Thus, a plethora of studies has been carried out to shed light on the dynamics of the aggregation, and the binding with the membranes [16,105,106,107,108,109,110,111,112]. As an example, cholesterol is one of the crucial lipid components in the cellular membranes. Its content can vary, and to date it is not clear how this is related to some of the diseases previously discussed. Its concentration in the membranes has been shown to trigger membrane damage. In fact, at a concentration below 30% in the lipid membrane, cholesterol represses the fibrillation and the penetration of Aβ_(1-40)_ in the membranes, whereas above this concentration, membrane damage occurs [113]. At a macromolecular lever, increasing cholesterol content leads to an increase in the membrane stiffness, which could affect the physical chemistry nature of this interaction. Additionally, in the raft-like model membranes, the presence of cholesterol accelerates amylin fibril growth and enhances the formation of pores in vitro [110]. Cholesterol also increases electrostatic interactions between charged residues and lipid head groups by fibril–membrane binding in the case of Aβ [114]. Of further concern is the Aβ, which has been proved that anionic lipids prompt the crystalline ordering of the peptide, favoring the β-sheet structure and consequently, the fibril formation [115]. 

**Figure 2:**
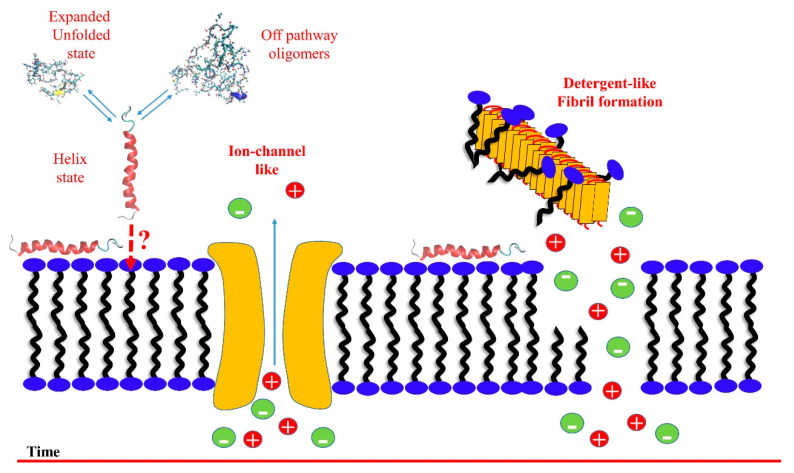
Schematic representation of the proposed two-step mechanism concerning the membrane disruption by hIAPP [5] and Aβ [7] through membrane fragmentation (detergent-like) and ion-channel-like pore formation. In the time scale, pore formation occurs before the detergent-like mechanism, where fibril grow on the bilayer’s surface by removing lipids from one leaflet (carpeting) and then ripping the remaining leaflet (detergent) with consequent membrane damage.

A more general two-steps mechanism for both hIAPP [5] and Aβ40 [7] was proposed, describing the early steps of the membrane damage, likely due to the oligomeric species, and showing some conventional features in membrane damage. Based on the dye leakage fluorescence kinetic experiments, in a first step, the two amyloidogenic proteins form ion-channel-like pores, whereas, in a second step, a detergent-like mechanism is detected, schematically illustrated in Figure 2. Thioflavin T (ThT) fluorescence kinetic experiments and NMR spectroscopy were used to detect the presence of fibrils, and to determine the solution’s intermediate species diffusion, respectively [116].

Furthermore, the conformational transition from random coil to, in most of the case, a helical-stabilized structure in the presence of the membranes, seems to be an essential prerequisite until the irreversible conversion to a β-sheet rich structure [40]. Specific and compelling case studies have been reported in the literature [57,60,69,117,118].

This issue has been extensively investigated. The outcomes seem leading to the certainty of interactions among amyloids and membranes. In fact, this has to be considered as a two-fold perspective. On the one hand, peptide aggregates may influence the chemical–physical properties of the membranes, e.g., permeability and rigidity. On the other hand, the lipid membrane may affect the unfolding and the aggregation processes [98,119,120,121,122,123,124]. 

Many experimental pieces of evidence support the hypothesis that a common mechanism of interaction may exist, shared among α-synuclein, Aβ, IAPP, and Prion oligomers towards the membranes [6]. One of the milestones is represented by the great work of Miranker et al., carefully summarized in their review [93], in which it is highlighted how membranes act as a catalyst in the amyloid formation, for all the Aβ [112,125], α-synuclein [98,126], and IAPP [97,117,121]. 

Intriguingly, as h-IAPP, Aβ, α-synuclein, and prion have been found to interact with bilayers through a pore formation mechanism [127].

However, we have recently shown that the phospholipids free in solution might mediate the membrane–protein interaction by forming a thermodynamically stable complex. This aggregate of unknown stoichiometry is considered to be the driving force of such interaction [128]. This study, based on a combination of experimental and computational approaches, is strongly supported by a previously published theoretical model [129].

Furthermore, preliminary results from our group have pointed out the concentration of free lipids in solution as a key factor in both the aggregation and the membrane damage processes. This aspect is true not only for the IAPP, but also for other amyloidogenic proteins, again suggesting that a common mechanism might exist.

### 3.2. Interaction with Small Molecules and Ions

The disruptive interaction with the membranes is a central event in the development of amyloidogenic diseases. Thus, inhibiting or preventing the membrane damage, together with the oligomerization and the fibril growth, might be one of the possible promising therapeutic strategies [130]. In this perspective, many studies aimed at investigating of the role of small molecules, as well as the interactions with physiologically relevant ions, which have been widely studied, such as calcium, copper or zinc [94,95,104,131,132], since the deep understanding of these mechanisms might be of fundamental importance.

It is useful to categorize these molecules into two different classes, based on the action mechanism: the ones acting on the oligomer species and the β-breakers; those that disrupt the “β-symmetry,” required for the toxicity and associated to fibrillar structures, blocking this abnormal conformational change. As an example, resveratrol, part of the family of polyphenols, has been found to inhibit either the aggregation of hIAPP and its interaction with negatively charged membranes [101,102]. 

Furthermore, this molecule rearranges Aβ_(1-42)_ soluble oligomers, fibrillar intermediates, and amyloid fibrils into a non-toxic, high molecular weight and unstructured alternative species [133]. Concerning the same protein, it has been found that orcein derivatives accelerate the fibrillogenesis, significantly reducing the concentration of toxic oligomers [134]. 

To date, cyclodextrins, lacmoid, Congo red, surfactants (SDS and LiDS), polyamines, and affibody are only a few of the classes of molecules interacting with the Aβ amyloid peptide [135]. Additionally, the interaction between the protein and CdTe NPs has been studied using different experimental approaches [136,137], as well as the effect of the silybin B as an inhibitor of the amyloid growth and toxicity [138].

Interestingly, in some cases, it is the combined effect between the small molecule and metal ions, which perturbs the amyloidogenic properties [132,139,140,141]. 

The understanding of this issue at a molecular level represents a step further in the possible future design and development of a drug against the pathologies as mentioned earlier.

## 4. Lipid-Assisted Protein Transport

Many researchers over the past thirty years have described the molecular mechanism of IDPs toxicity. As previously mentioned, the toxicity of amyloid proteins is often linked to membrane damage-associated mechanisms. However, the molecular details of the membrane disruption processes are still unknown. Currently, the most confirmed hypotheses include (i) the generation of stable transmembrane protein pores (toxic oligomer hypothesis), (ii) membrane destabilization via a “carpet model,” (iii) lipid extraction by amyloids via a “detergent-like” mechanism, and (iv) membrane damage by lipid peroxidation (inflammation/oxidative stress hypothesis). Either way, it is still not yet clear whether these models are mutually exclusive or if (and how) they cooperate to trigger membrane damage. The majority of the literature focuses on lipid–protein interactions occurring at the bilayer, rather than interactions in the aqueous phase. However, a chemical equilibrium exists between monodispersed lipids and their self-assembled species, dependent on their respective critical micellar concentrations (CMCs). The CMC is a function of the net charge in the polar head and of the number of carbon atoms composing the hydrophobic chain. In some cases, e.g., for molecules with short lipid tails (less than or equal to 14 carbon atoms per lipid chain) or negatively charged, the concentration of free lipids in the aqueous phase may reach values in the µM range [128]. In contrast, long lipid tailed zwitterionic lipids have a CMC around the nM range [128,142]. Since proteins are also present at µM concentrations in most of the amyloid/membrane interactions assays, it is plausible that a lipid–protein binding equilibrium exists in the aqueous phase, which might influence their membrane insertion. 

Recently, a phenomenological model based on experimental findings was proposed to simulate the transfer kinetics of a lipid–protein complex from water to the lipid-bilayer phase [129]. We found out that the water-soluble lipid–protein complex inserts into the membrane faster than the free protein due to the hydrophobic differences between the lipid–protein complex and the bare protein. This model is supported by several simulations and kinetic experiments carried out on hIAPP. Particularly, to study the influence of the free lipid concentration in fibril growth and membrane disruption, the ThT and dye-leakage assay were used, respectively. Importantly, our results offer a novel mechanistic explanation as to the reason why the bilayer thickness is inversely correlated to the membrane damage induced by amyloid proteins, as shown by some experimental pieces of evidence previously published [143]. A general mechanism of lipid-assisted amyloid penetration into the membranes able to explain toxicity/membrane damage of hIAPP has been proposed. Free phospholipids in the aqueous phase are in chemical equilibrium with the phospholipids self-assembled into membrane bilayers and by forming a lipid–protein complex able to transport hIAPP into the core of the bilayer [128]. This finding has been proved by using either computational and experimental approaches. In particular, the lipid–protein complex has a two-fold implication. On the one hand, it triggers the interactions with the membrane, due to the increasing global hydrophobicity. On the other hand, the self-assembly of the protein is perturbed. Lipids with high CMCs actively repress fibril formation. A systematic work has been carried out, investigating a wide range of CMCs. The relevant main conclusions suggest that the free phospholipid concentration in the aqueous phase acts as a switch between the ion-channel-like pore formation and the detergent like-mechanism. In particular, phospholipids having high CMCs (order of µM) favor ion-channel-like pores, and at the same time, repress fibril formation in the aqueous phase. Vice versa, low CMC phospholipids (order of nM) repress pore formation and favor the detergent-like mechanism. 

**Figure 3:**
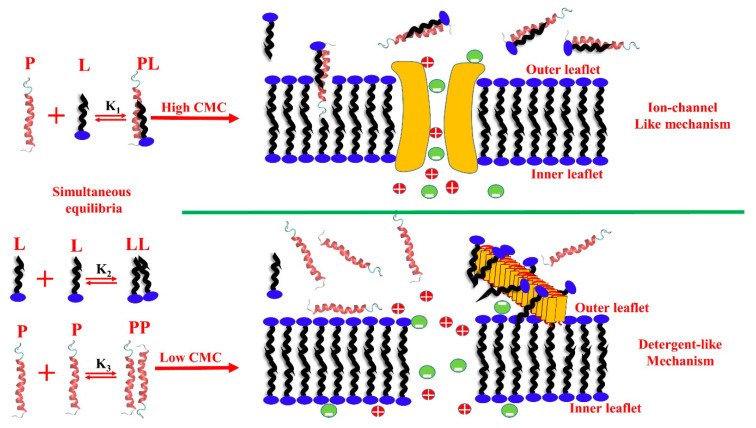
Schematic representation of free lipids in the aqueous phase, interacting with phospholipid large unilamellar vesicles (LUVs) having different critical micellar concentrations (CMCs). In the aqueous phase containing lipids and proteins, three simultaneous and competing equilibria should be considered, lipid–lipid (equilibrium constant of K_2_), protein–protein (equilibrium constant of K_3_), and lipid–protein (equilibrium constant of K_1_) complex equilibria. Fixed protein concentration and high phospholipid concentration favor lipid–protein complex formation. In the presence of high CMC phospholipids (short hydrocarbon tails), hIAPP form a lipid–protein complex, penetrate the membranes by forming ion-like pores and do not form fibrils. hIAPP in the presence of low CMC phospholipids do not form lipid–protein complexes, and only a detergent-like mechanism is detected [128].

Figure 3 schematically summarizes the above discussed concept.

Moreover, to investigate the role of the thickness of the bilayer, the same fluorescent kinetics experiments have been performed using the LUVs having lower CMCs in the presence of free phospholipids possessing higher CMCs. The CMCs values are strictly related to the length of the hydrophobic chain, thus to the thickness of the bilayer. In particular, the LUVs used in these experiments was also the thicker one. No detergent-like mechanism and no fibril formation were observed for this system. Vice versa, analogously to the results obtained for the higher CMCs system, pores were detected. The lack of the detergent-like mechanism does not depend on the mismatch between the thickness of the bilayer and protein lengths, but it is strongly linked to the concentration of free lipids. 

Finally, additional biophysical investigations were carried out in our lab to study whether this behavior was a feature of h-IAPP or the concept of a lipid–protein chaperone can be extended to Aβ and α-synuclein as well. Interestingly, the preliminary results agree with the hypothesis previously described: the chemical equilibria involved in these complex interactions are all related to each other. Therefore, the lipidic CMC affects both the protein aggregation and the associated interaction with membranes. Our results suggest that a shared mechanism, involving some of the amyloidogenic IDPs under investigation, might exist. 

## 5. Symmetry-Breaking Transitions of Oligomers Self-Assembling

The self-assembly of amyloidogenic proteins in the aqueous phase occurs through a series of sequential processes and molecular events. Monomers self-assemble into transient oligomeric aggregates and evolve into fibrils, non-crystalline structures abundant in beta-sheets. The molecular structure of the fibrils was extensively studied mainly by electron microscopy (EM) [144], NMR [83,145,146,147,148], and cryo-EM studies [149,150,151]. Unfortunately, X-ray crystallography studies cannot provide atomistic details on fibrillar aggregates due to the lack of a long-range order, strictly necessary to observe Bragg diffraction. Recently, short-range order and the presence of α-helix structures have been observed through the tip-enhanced Raman spectroscopy technique (TERS) [152]. The characterization of oligomeric structures is not an easy goal to achieve [137], due to their inherent transient nature. Recently, a theoretical model, supported by extensive molecular dynamics simulations and experiments, has been developed. It is based on the description of the intrinsic asymmetry in protein aggregates using models of field theory [153]. In its most straightforward formulation, the theory considers a linear aggregate of polypeptides (proteins), which can have different conformational states, and interact through intermolecular forces that depend on the internal conformational states. 

This model predicts, under particular conditions, a spontaneous transition from a homogeneous state (an equally spaced protein array) to a state of spatially modulated structures. It has been proved that this transition may occur if the proteins possess conformational flexibility, namely two interchangeable conformational states. An ordered state (A), given by the sum of amino acids in α-helix and β-sheet structures, and the other disordered state (B), as a consequence of the total number of amino acids in turn and coil conformations, of comparable energy. 

The Gibbs’ free energy of array subunits of proteins has been partitioned into three contributions: (i) the mixing entropy, (ii) the self-energy measuring the conformational stability due to intramolecular bonds and solvent interactions (corresponding also to the unfolding energy with the sign changed), and (iii) the conformation-depending energy, including for simplicity the nearest-neighbor protein interaction energy alone.

The mathematical model leads us to the following main conclusions. If a symmetric array of oligomers that can adopt two different geometries and becomes unstable, a spontaneous breaking occurs, and the array assumes alternating A and B configurations. Instability occurs if (i) the internal energies of the arrangements A and B differ in the order of a few K_B_T (about 1 kcal mol^−1^), (ii) if there is a substantial geometric difference between the A and B conformations, and (iii) if there is significant interaction energy between the oligomers (of the order of 10 K_B_T). The above described energy scenario suggests that assemblies of amyloidogenic IDPs are the best candidates to observe the conformational instability previously described in this chapter. Tiny energy fluctuations may trigger the formation of oligomeric patterned aggregates. MD simulations repeatedly observed the predicted behavior in Aβ, hIAPP, compounds of Aβ and hIAPP, and the Prion. In these cases, the self-adhesion energy is larger than the unfolding energy [154], as predicted by the model. Moreover, this theory justifies the formation of multimeric proteins. For instance, in their native state, monomeric blue copper proteins plastocyanin [155], amycianin [156], and azurin [157,158], have large unfolding energy and negligible adhesion energy. Moreover, other well characterized monomeric proteins, such as cytochrome [159] or ubiquitin [160], exhibit similar behavior, detected using differential scanning calorimetry [161,162] and negligible adhesion energy. This is probably the reason why these proteins in their native state exist as monomers. In contrast, dimeric wt-SOD [163], apo-SOD [164], and IDPs [154] possess exiguous unfolding energy and high adhesion energy. Thus, they form oligomers with an alternating arrangement of the individual building blocks (monomers). Another unique system that has to be considered is the G37R-SOD [165] mutant, associated with familial amyotrophic lateral sclerosis (FALS). The replacement of the amino acid R with G results in the loss of a hydrogen bond, topologically on the opposite side, with respect to where the Zn^++^ and Cu^++^ ions are allocated corresponding to a significant contribution to the thermodynamic stability of SOD. The consequence of the loss of the hydrogen bond involves the loss of the two metal ions, and therefore, a drastic decrease in unfolding energy. The mutant G37R-SOD, for example, simultaneously unfolds and dissociates due to the loss of the thermodynamic stability of the individual monomers.

## 6. Common Features, Apparent Irreproducibility and Paradoxes, A Brief Overview

For 30 years, plenty of biophysics studies on IDPs have been published, and it has been shown that this family of proteins share many physical–chemical characteristics. Although they have different amino acid sequences, they show very similar dynamics and structural behaviors; indeed, they have also been named chameleonic [153,166] proteins. In an aqueous solution, they form non-crystalline-structured aggregates, rich in beta-sheet structures, penetrate model membranes by forming pores, and damage the membrane with a mechanism described as detergent-like.

Crucial features of the amyloidogenic IDPs are the misfolding and their subsequent capability to self-associate. This process can be theoretically predicted considering the energy scenario related to the system, particularly the patterned array of ordered and disordered conformers, originating from a configurational instability. The symmetry-breaking theory justifies not only the self-assembling into an oligomeric structure, but also the behavior of multimeric proteins [153]. 

Moreover, IDPs’ toxicity, both in vitro and in vivo, has been associated with their ability to damage membranes. The apparent irreproducibility of the data reported on IDPs’ aggregations have been attributed to the amphiphilic character of these proteins, since they show a CMC [167], exactly as phospholipids do. For instance, on the one hand it is well known that rIAPP is not toxic to cultured cells and does not form fibrillary aggregates. On the other hand, studies on rIAPP, interacting with negatively charged membranes, have shown that this protein can damage the membrane bilayers [35,168]. However, this behavior has not been observed in zwitterionic membranes interacting with rIAPP [5]. In addition, it has also been reported that rIAPP at concentrations higher than 20 µM is toxic for cultured cells [169]. If the hypothesis that toxicity is associated with membrane damage is correct, this behavior of rIAPP appears to be a paradox. To date, two hypotheses have been proposed to explain the in vivo and in vitro data: the amyloid hypothesis and then the toxic oligomers hypothesis. These two hypotheses have proved insufficient to explain the observed experimental facts. As a matter of fact, the inability to develop any drugs capable of blocking the IDP-related diseases clearly shows that to date, the mystery of IDPs’ toxic action has not been solved. Recent biophysics investigations support the hypothesis that free phospholipids may act as a chaperone for IDPs during their interaction with the membrane. All of this could give a new impetus in solving the open mystery of amyloidogenic proteins. The hypothesis at the basis of the model is that free phospholipids can form a stable complex with amyloidogenic proteins, which may explain the apparent paradox of rIAPP. 

The law of chemical equilibrium regulates lipid–protein complex formation; therefore, the lipid–protein complex is favored by an increase in lipid or protein concentration. Lipids with high CMCs favor the formation of the complex and therefore, the transfer into the bilayer, as previously proposed for hIAPP [128]. Thus, the rIAPP can form the lipid–protein complex in the presence of LUVs, containing lipids with a high CMC (charged lipids), while it cannot form the complex with the lipids characterized by a low CMC (zwitterionic lipids). Further studies carried out in our lab suggested that the latter might be a common mechanism shared by different IDPs. [170].

From the overall data reported in this review, an exciting scenario emerges: despite the different individual characteristics described in Section 2, hIAPP, Aβ, a-synuclein and prion show several common properties, regardless of their interactions with membranes. As a matter of fact, there are some evidences leading us to the hypotheses that the aforementioned interactions seem to have a predominant role in the insurgence of the related pathologies. For instance, all four proteins considered here can damage the membrane by forming pores, and the proteins are transported into the bilayer by free lipids in the aqueous phase. Since the pores’ formation was correlated with the toxicity of these proteins, the common characteristic suggests that the molecular mechanism of membrane damage may be shared. The fibrils formed by the four proteins tend to be predominantly rich in beta-sheets. Finally, the oligomers formed by hIAPP, Aβ and prion have a common characteristic: in the aqueous phase, they form ordered–disordered structures that are independent of the amino acid sequence of the protein considered. In conclusion, amyloidogenic proteins interacting with membranes have similar physical–chemistry features, which have to be carefully understood in order to entangle their complexity and shed light on the reasons at the base of their relation with the aforementioned pathologies.
